# Exosomes in Mastitis—Research Status, Opportunities, and Challenges

**DOI:** 10.3390/ani12202881

**Published:** 2022-10-21

**Authors:** Zhong-Hao Ji, Wen-Zhi Ren, Hong-Yu Wu, Jia-Bao Zhang, Bao Yuan

**Affiliations:** 1 Department of Laboratory Animals, College of Animal Sciences, Jilin University, Changchun 130062, China; 2 Department of Basic Medicine, Changzhi Medical College, Changzhi 046000, China; 3 Jilin Academy of Agricultural Sciences, Jilin City 132101, China

**Keywords:** mastitis, exosomes, miRNA, biomarkers, drug delivery vehicles, intercellular communication

## Abstract

**Simple Summary:**

Mastitis is one of the most common diseases in dairy cows, causing large economic losses. The development of early diagnostic markers for mastitis and the elucidation of pathogenesis are of great importance for the prevention and treatment of mastitis. Exosomes are extracellular vesicles secreted by cells that contain cargoes of nucleic acids and proteins that reflect the state of the cell and mediate intercellular communication. In this review, we introduce the knowledge of exosomes, review the current status of exosome research related to mastitis, and look forward to the application of exosomes in the diagnosis and treatment of mastitis and in performing disease mechanism elucidation.

**Abstract:**

Mastitis, which affects milk quality and yield, is one of the most common diseases in dairy cows, causing large economic losses. Cow mastitis is classified into clinical and subclinical types. Subclinical mastitis presents without obvious lesions in the udder or noticeable change in milk samples, indicating persistent chronic infection that is difficult to detect and treat. Therefore, finding specific biomarkers is of great significance for the early diagnosis and treatment of subclinical mastitis. As mediators of intercellular communication, exosomes have been shown to be extensively involved in various physiological and pathological processes in the body. Exosomes in milk, blood, and cell supernatant can carry stable cell source-specific nucleic acids, proteins, and metabolites. Hence, exosomes show great application prospects for early diagnosis, targeted therapy, and disease mechanism analysis. In this review, we summarize the biogenesis, biological functions, and methods of isolating and identifying exosomes and review the current status of exosome research related to mastitis. Finally, in view of the application of exosomes to diagnose, treat, and perform disease mechanism analysis in mastitis, deficiencies in recent research on mastitis exosomes are described, and the direction of future exosome research efforts in mastitis is proposed.

## 1. Introduction

Mastitis affects milk quality and yield and is one of the most common diseases in dairy cows, causing large economic losses [1,2]. One study showed that approximately 60–70% of all antimicrobials used on dairy farms are used to prevent and treat mastitis [3], which raises concerns about antibiotic residues, resistance, and food safety [4]. Cow mastitis can usually be classified into two categories, namely, clinical mastitis and subclinical mastitis. The main pathogen in clinical mastitis is gram-negative bacteria represented by *Escherichia coli*, and its clinical characteristics are severe inflammatory reactions, abnormal milk properties, and mammary gland redness and swelling [5]. Subclinical mastitis is also called recessive mastitis, and the main pathogens are gram-positive bacteria represented by *Streptococcus* spp. and *Staphylococcus* spp. [6,7,8]. It is characterized by a weaker inflammatory response than observed in subclinical mastitis, persistent chronic infection, decreased milk production, and increased somatic cell count (SCC), but there are no obvious lesions in udder or milk samples [4,9]. Finding specific diagnostic markers is of great significance for the early detection and treatment of subclinical mastitis. In addition, multiple in vitro experiments with bovine mammary epithelial cells have shown that *E. coli* and *Staphylococcus aureus* mediate immune and inflammatory responses through different molecular mechanisms [10,11,12]. Therefore, understanding the host’s immune and inflammatory response mechanisms triggered by infection is particularly important for the prevention and treatment of mastitis.

**Figure 1:**
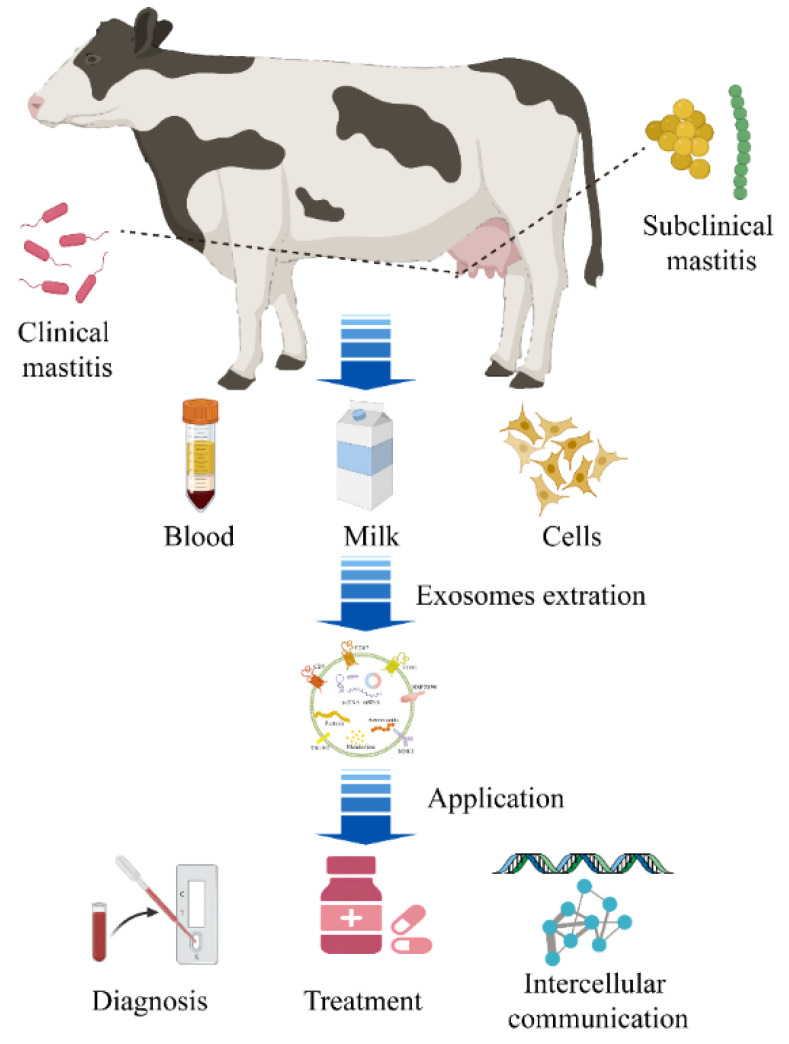
Mastitis-related exosomes for diagnosis, treatment, and disease mechanism analysis. The main pathogen in clinical mastitis is gram-negative bacteria represented by *Escherichia coli*, and the main pathogens in subclinical mastitis are gram-positive bacteria represented by *Streptococcus* spp. and *Staphylococcus* spp. Exosomes have been found in milk, blood, and cell supernatant, and nucleic acids, proteins, and metabolites encapsulated by exosomes are source-specific and stable. In mastitis, exosomes show great application potential for early diagnosis, targeted therapy, and disease mechanism analysis.

The mammary gland is a secretory organ enriched with vasculature. Mammary epithelial cells are the main functional secretory cells in the mammary gland and share certain characteristics with immune cells. Notably, studies have shown that exosome-mediated intercellular communication is widely involved in physiological and pathological processes [13,14]. Exosomes have been found in milk, blood, and cell supernatant, and nucleic acids, proteins, and metabolites encapsulated by exosomes are source-specific and stable. Exosomes show great application potential for early diagnosis, targeted therapy, and disease mechanism analysis. In this review, we first summarize the biogenesis, biological functions, isolation, and identification methods of exosomes. Second, the research status of mastitis-related exosomes, including mainly mammary epithelial cell-derived exosomes and milk-derived exosomes, is reviewed. On this basis, the potential predictive and diagnostic biomarkers of mastitis found in these studies are summarized, and the roles played by exosome-mediated intercellular communication in the pathogenesis of mastitis are analyzed. Finally, in view of the application prospects for exosomes to the diagnosis, treatment, and disease mechanism analysis of mastitis, deficiencies of the recent mastitis exosome research are analyzed, and future directions for mastitis exosome research are proposed (Figure 1).

## 2. Introduction to Exosomes

### 2.1. The Biogenesis of Exosomes

**Figure 2:**
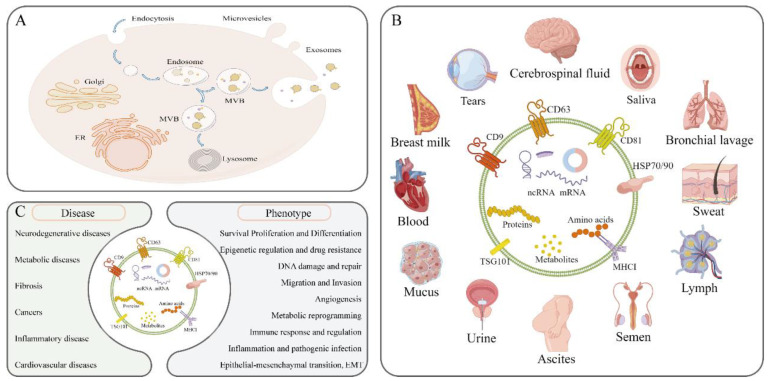
Biogenesis and biological functions of exosomes; (**A**) Exosomes passing through the invagination of the plasma membrane are released through the intracellular multivesicular pathway; (**B**) Exosomes are found in various bodily fluids and contain cell-derived membrane proteins, cytoplasmic proteins, nuclear proteins, mRNAs, noncoding RNAs (ncRNAs), and various metabolites; (**C**) Exosomes play a role in various diseases and phenotypes.

Almost all cells secrete extracellular vesicles, which are roughly classified into two categories: ectosomes and exosomes [15]. Ectosomes are directly produced by externally budding plasma membrane, and the diameter of ectosomes is between 50 nm and 1 µm [16]. In contrast, the diameter of exosomes is approximately 40–160 nm, and exosomes are derived from invagination of the plasma membrane and the formation and release of endosomes and intracellular multivesicular bodies (MVBs). The Golgi apparatus and endoplasmic reticulum promote the formation of endosomes, which mature and ultimately produce MVBs, which can either fuse with lysosomes and be degraded or fuse with the plasma membrane and release the internal intraluminal vesicles (IVLs) in the form of exosomes [17,18] (Figure 2A).

A variety of proteins are involved in the origin and biogenesis of exosomes. Rab GTPase protein controls endosomal trafficking [19], with RAB27A and RAB27B playing roles in MVB docking with the plasma membrane [20]. KIBRA has been reported to inhibit the proteasome degradation of RAB27A, controlling exosome release [21]. The mammalian endosomal sorting complexes required for transport (ESCRT) system consists of ESCRT-0, ESCRT-I, ESCRT-II, ESCRT-III, VSP4-VTA1, and ALIX homodimers [22], including tumor susceptibility gene 101 (TSG101), hepatocyte growth factor-regulated tyrosine kinase substrate (HRS), signal transducing adapter molecule 1(STAM1), apoptosis-linked gene 2-interacting protein X (ALIX), and other protein subunits [23]. Among these complexes, ESCRT-0, ESCRT-I, ESCRT-II, and ESCRT-III are involved in the formation of IVLs. ESCRT-0, ESCRT-I, and ESCRT-II may be involved in cargo sorting, while ESCRT-III is involved in membrane deformation and fission [24,25,26]. However, a recent study reported that RAB31 marks and controls an ESCRT-independent exosome biogenesis pathway [27]. The tetraspanins CD9, CD63, and CD81 and the lysosome-associated membrane proteins LAMP1 and LAMP2 prevent MVBs from degradation by lysosomes [17], while neutral sphingomyelinase 2 (nSMase2) promotes vesicle formation [28]. In this respect, a commercial reagent target nSMase2, GW4869, is widely used in exosome-related research [29,30].

In addition, in terms of the exosome-cargo-sorting mechanism, hnRNPA2B1 [31], RBMX (recognition motif CCAU) [32], FMR1 (recognition motif AAUGC) [33], Alyref and Fus (recognition motif CGGGAG) [34], Cx43 [35], MEX3C [36], YBX-1 [37], Lupus La [38], and other RNA-binding proteins recognize microRNAs (miRNAs) arr harboring specific motifs and load these miRNAs onto exosomes. In conclusion, exosomes biogenesis and cargo-loading process exhibit high heterogeneity and great complexity, and continuous efforts are needed to characterize these mystical mechanisms.

### 2.2. Biological Functions of Exosomes

Exosomes can be isolated from various bodily fluids, such as cerebrospinal fluid, tears, breast milk, blood, mucus, urine, ascites, semen, lymph, sweat, bronchial lavage, and saliva [39,40,41,42] (Figure 2B). The contents of exosomes include a variety of cell-derived biomolecules, such as membrane proteins, cytoplasmic proteins, nuclear proteins, mRNAs, noncoding RNAs (ncRNAs), and various metabolites [43].

The extensiveness of exosome sources and the richness of exosome contents determine their functions, which are diverse. Tumor cell-derived exosomes are widely involved in the occurrence and development of cancer [44], and affect tumor microenvironment remodeling [45], angiogenesis [46], tumor cell migration and invasion, and epithelial-mesenchymal transition (EMT) by mediating intercellular communication [47], drug resistance [48,49], and resistance to cell death [50]. In neurodegenerative diseases such as Alzheimer’s disease (AD) and Parkinson’s disease (PD), miRNAs in exosomes derived from peripheral blood and cerebrospinal fluid have shown promise as preclinical diagnostic markers [51,52,53,54]. Moreover, adipose stem cell-derived exosomes exerted neuroprotective effects on AD in a rat model [55]; type Ⅱ macrophages (M2) and microglia-derived exosomes alleviated neuronal damage and mitochondrial damage in AD, which was mediate through PINK1/Parkin pathway dysfunction [56]; human amniotic fluid mesenchymal stem cell-derived exosomes exerted neuroprotective effects by inhibiting the inflammatory response of microglia [57]; and bone marrow mesenchymal stem cell-derived exosomes alleviated microglia dysfunction in PD by inhibiting Sp1 signaling activation and neuronal apoptosis [58]. In cardiovascular disease, drug-pretreated mesenchymal stem cell-derived exosomal miR-146a-5p promoted cardiac repair [59]. In inflammatory diseases, such as inflammatory bowel disease (IBD) and acute kidney injury (AKI), bone marrow mesenchymal stem cell-derived exosomes have been shown to elicit certain outcomes. For example, exosomes carrying miR-539-5P alleviated IBD by inhibiting pyroptosis [60], and lipopolysaccharide (LPS) induced renal tubular epithelial cell-derived exocrine miRNA-19b-3p release through exosomes, which drove renal injury by promoting M1-type macrophage polarization [61]. In polycystic ovary syndrome, follicular fluid-derived exosomal miR-143-3p/miR-155-5p affected follicular development by regulating glycolysis [62]. In liver fibrosis, exosomes derived from macrophages and Kupffer cells mediated the occurrence and development of fibrosis by regulating the EMT of epithelial cells and activation of hepatic stellate cells [63,64]. In conclusion, numerous studies have demonstrated the roles played exosomes in a variety of diseases and phenotypes (Figure 2C).

### 2.3. Isolation and Identification of Exosomes

Based on the physicochemical properties of exosomes, six strategies for exosome isolation with different characteristics have been reported thus far, including ultracentrifugation, ultrafiltration, immunoaffinity capture, polymer precipitation, size exclusion chromatography, and microfluidics [65]. Among these strategies, ultracentrifugation is the gold standard and the most commonly used method for exosome isolation. An ultracentrifuge capable of reaching 150,000× *g* centrifugal force is necessary for performing this method. The advantage of ultracentrifugation in separating exosomes relates to its suitability for use in various complex bodily fluid and large-volume samples, low cost, and low risk for contamination. The disadvantages include high-cost hardware, the process is difficult and time consuming, and it is not suitable for small samples. In addition, protein aggregates caused by extreme dissociation may exert unknown effects on the quantification and subsequent functional analysis of exosomes isolated by ultracentrifugation [66].

Polymer precipitation is another commonly used technique for exosome isolation [67], and the hydrophilic polymer polyethylene glycol (PEG) [68] is widely used in this process. This strategy is the basis of several commercial exosome isolation kits, such as the Total Exosome Isolation Reagent (Invitrogen, Carlsbad, CA, USA), ExoQuick (System Biosciences, Palo Alto, CA, USA), Wayen exosome isolation kit (WY, Shanghai, China), Ribo exosome isolation reagent (Ribo, Guangzhou, China), and miRCU exosome kit (Qiagen, Hilden, Germany). Using a kit to extract exosomes does not require special equipment and shows a wide range of applications. A large number of exosomes can be isolated from urine, cell supernatant, blood, ascites, and other bodily fluids in a short time by following simple and easy-to-follow instructions [67,69,70]. However, the stability and purity of the exosomes extracted by these kits need to be considered. In one study, researchers compared exosomes isolated by five kits with exosomes isolated by ultracentrifugation, characterizing technical stability, and reliability in terms of vesicle number, purity, and integrity of RNA in contents; the results showed a high degree of variability in the polymer precipitation-based exosome isolation methods [71]. Therefore, careful consideration is needed when choosing an exosome extraction method.

After selecting the appropriate method to isolate exosomes based on sample characteristics and laboratory conditions, the next step is identification of the isolated exosomes. In 2014, the International Society for Extracellular Vesicles (ISEV) [72] proposed that the isolated exosomes need to be identified through three processes [73,74]: (1) Western blotting (WB) to identify exosome surface markers, such as the positive markers CD9, CD63, CD81, TSG101, ALIX, and HSP70, and the negative markers CYCS, Calnexin, GPR94; (2) transmission electron microscopy (TEM) to identify the morphology of exosomes, which form a cup-like shape and a double-layer membrane structure; and (3) nanoparticle tracking analyzer (NTA) detection is used to determine endosome size and concentration. Compared with the other characterization methods, NTA technology involves the simplest preparation and can better distinguish the original state of exosomes. In addition, exosomes are tracked with the commonly used fluorescent dyes PKH-67, PKH-26, DIO, DID, DIL, CFSE, and calcein-AM.

## 3. Exosomes Associated with Mastitis

### 3.1. Milk-Derived Exosomes

Milk contains many exosomes, and the content and biological functions of milk-derived exosomes change during mastitis. Sun and Cai et al. established the miRNA expression profile of bovine milk exosomes in response to *S. aureus* infection, and they identified bta-miR-142-5p and bta-miR-223 as possible biomarkers for the early diagnosis of mastitis [75,76]. Ma et al. generated miRNA expression profiles of milk exosomes under normal conditions and after *S. aureus* infection and verified the targeting of bta-miR-378 and bta-miR-185 to specific genes [77]. Compared with sampling analysis at a single time point after infection, the results of continuous multipoint sampling analysis better characterize the specificity of exosomes and their internal cargo in mastitis milk. Analysis of subclinical mastitis milk exosomes collected for three consecutive days by Mara et al. showed that the size and concentration of milk exosomes, as well as the miRNA cargo inside them, remained very stable over the course of three days. The analysis revealed that specific miRNAs in milk exosomes were associated with certain physiological states, and the highly expressed miRNA bta-miR-223-3p showed the potential to be used as an early diagnostic marker for subclinical mastitis [78]. In addition, numerous studies have characterized the expression of glycoprotein [79], proteins [80,81], miRNAs [82], and long noncoding RNAs (lncRNAs) [83] in normal milk exosomes using omics technology. Wang et al. characterized the expression of miRNA in milk exosomes under heat stress [84]. Monica Colitti examined miRNA expression and regulatory patterns from milk exosomes of cows experienced group relocation [85]. Selçuk Özdemi identified and characterized the exosomal miRNA in the milk and colostrum of Holstein and Doğu Anadolu Kirmizisi (DAK) cows [86]. These examples suggest that the milk derived exosomes cargo is not only affected by local inflammation, but also responds to environmental changes or stress.

In general, most of the studies related to milk-derived exosomes in mastitis are presented in miRNA atlases. These results are the basic data for studying the mechanism of mastitis infection and screening early diagnostic markers, and they have important reference value. However, the characterization of the biological functions of exosomal cargo needs to be confirmed in follow-up analyses.

### 3.2. Mammary Epithelial Cell-Derived Exosomes

Bovine mammary epithelial cells (BMECs) were isolated from bovine mammary gland tissue and cultured in vitro, and they showed limited passaging ability. MAC-T cells are a bovine mammary epithelial cell line widely used in mastitis-related research [87,88]. Mojisola Ogunnaike et al. showed that the miRNA and protein profiles in MAC-T-cell-derived exosomes were consistent with those of exosomes derived from milk, and hence, MAC-T cells can be powerful tools for milk exosome-related research [89]. Zhang et al. analyzed major protein species and biological processes involved in ultracentrifuged BMEC-derived exosomes by mass spectrometry [90]. Yu et al. generated expression profiles of mRNA and lncRNA in normal and *S. aureus*-infected MAC-T-cell-derived exosomes [91].

Selenium is an essential trace element in the body and exhibits various physiological functions, such as anti-inflammatory and antioxidant activities. Many clinical and basic studies have shown that selenium may relieve bovine mastitis by inhibiting the NF-κB and MAPK signaling pathways or activating NRF2 [92,93,94,95,96]. To further explore the molecular mechanism by which selenium alleviates mastitis, Jing et al. analyzed the effect of selenium on exosomal RNA derived from MAC-T cells and determined the mRNA levels in exosomes derived from selenium-pretreated normal cells and from cells that had been infected with *S. aureus* [97].

As the main virulence factor of gram-positive bacteria, Lipoteichoic acid (LTA) has been mainly used to create models for the study of subclinical mastitis. Studies have reported that LTA-treated HC11 mouse mammary epithelial cell- and MAC-T cell-derived exosomes induced Macrophages type Ⅰ(M1) polarization to promote inflammatory responses [98,99]. While exosomes secreted by mammary epithelial cells exerted paracrine effects that mediated intercellular communication, these cells additionally regulated the proliferation, apoptosis, and inflammatory response of mammary epithelial cells in an autocrine manner [100]. Stimulation of mammary epithelial cells by LPS, a major virulence factor in gram-negative bacteria, resulted in increased levels of miR-193b-5p in exosomes [101]. Their roles and functions of exosomes derived from mammary epithelial cells in bacterial-induced mastitis have been initially explored. However, compared with other inflammatory diseases, such as acute nephritis and IBD, the depth and breadth of the research into mastitis need to be increased. Specifically, research into the sorting mechanism of exosome cargo in donor cells and the establishment of new exosome-mediated cell interaction models are needed.

### 3.3. Peripheral Blood and Urine Exosomes

Molecules in peripheral blood and urine have diagnostic value for various diseases. However, few studies have been conducted on peripheral blood and urine-derived exosomes with respect to mastitis, with most directly analyzing cargo changes in normal and mastitis samples. Li et al. analyzed miRNAs in the peripheral blood of healthy and mastitic cows, and 173 miRNAs were found to be differentially expressed; the results of a functional enrichment analysis showed that these miRNAs were mainly associated with the chemokine signaling pathway of the immune response [102]. Lai et al. analyzed the data of 11 normal cows and 15 cows with mastitis, and the results showed that the expression of miR-21 was significantly increased in the serum of cows with mastitis [103]. Dolma et al. established a mastitis model by injecting *S. aureus* and *E. coli* into bovine mammary glands and constructed miRNA expression profiles of peripheral blood 0, 1, 3, 5, and 7 days after injection. The results showed the high expression of miR-320a, miR-19a, and miR-19b, and low expression of miR-143, miR-205, and miR-24, with these sets of miRNAs playing important roles in *S. aureus*-induced mastitis. In addition, bta-miR-182 and the newly discovered conserved miRNA_15_7229 may be involved in the immune process in the late stage of *E. coli*-induced mastitis. In addition, miR-1301 was significantly upregulated, and miR-2284r was significantly downregulated in peripheral blood at different times after *S. aureus* infection, suggesting that these two miRNAs may be peripheral blood biomarkers of *S. aureus*-induced mastitis [104,105]. The research team Burim et al. collected bovine blood and urine samples at five time points 8 and 4 weeks before delivery, during the week of subclinical mastitis diagnosis, and 4 and 8 weeks after delivery. Via gas chromatography with Gas Chromatography-Mass Spectrometer (GC-MS) or Liquid Chromatography-Mass Spectrometry (LC-MS) methods, the metabolites were analyzed, and the results showed that specific metabolites in blood and urine showed high predictive and diagnostic ability for subclinical mastitis [106,107,108]. In view of the specificity and stability of exosomes, follow-up studies performed to analyze the sensitivity and specificity of the cargo in serum exosomes may determine their applicability for the prediction and diagnosis of mastitis and whether the cargo attenuates mastitis.

### 3.4. Mammary Tissue Exosomes

Mastitis is a disease mainly caused by bacterial infection, and the damage site is mainly the mammary tissue. The establishment of a transcriptional map of mammary tissue after mastitis infection is of great significance for the study of the pathogenesis and the development of biomarkers. Studying nephritis, some researchers have analyzed the molecular mechanism of kidney tissue-derived exosome-mediated diseases [61]. However, studies related to mammary tissue-derived exosomes in mastitis have not been performed to date. Li et al. analyzed the effect of *S. aureus* infection on the miRNA expression profile of mammary tissue. Compared with the number in the control group, 77 miRNAs were differentially expressed in the *S. aureus* infection group. Preliminary screening identified miR-223, miR-132 and miR-1246 as possibly mediating immune responses in bacterial infection, but to determine their specific functions in mastitis, further study is required [109]. Wang et al. simultaneously analyzed the changes in miRNA and mRNA expression profiles in mammary tissue infected by *S. aureus* infection and obtained 77 differentially expressed miRNAs and 1625 differentially expressed mRNAs [110]. Dolma et al. constructed the miRNA expression profile of mammary gland tissue after infection with *E. coli* or *S. aureus* and analyzed the similarities and differences in infection induced by the two types of bacteria. Preliminary screening revealed that miR-202 and miR-2357 may be biomarkers for clinical mastitis and that bta-miR-7863 may be a biomarker for subclinical mastitis [111]. Park et al. analyzed the effect of *Streptococcus agalactiae* infection on the miRNA expression profile of mammary tissue and identified 35 differentially expressed miRNAs, of which miR-223 was the most significantly upregulated and miR-26a was the most significantly downregulated [112]. At this stage, mammary tissue-related research focused on the work presented in miRNA atlases. Considering the cellular heterogeneity of mammary tissue, subsequent attempts using flow sorting, single-cell sequencing or spatial transcriptome sequencing is needed to improve the resolution of atlas analyses. Table 1 presents summarized studies related to mastitis-associated exosomes and includes information on sample sources, main experimental results, and methods of exosome isolation and identification.

## 4. Research Status of Exosomes That Are Predictive and Diagnostic Markers in Mastitis

Studies have shown that miRNAs in human milk are mainly derived from mammary epithelial cells, with peripheral circulating cells contributing relatively little [113]. Furthermore, in a study of two breeds of dairy cows, Holstein and Normand, which show different milk-producing characteristics, high-throughput sequencing revealed breed-specific expression profiles of miRNAs in milk [114]. Therefore, miRNAs in milk show the potential to be biomarkers of lactation performance and health status. During lactation, mammary gland cells undergo anabolism, consuming a large amount of energy and basic raw materials, which are derived mainly from blood. Mastitis affects tight junction proteins in cells, resulting in impaired blood–milk barrier integrity. Therefore, small-molecule metabolites [106], nucleic acids [103], and proteins [115] in blood show certain mastitis diagnostic efficacy. By analyzing the correlation between miRNA expression and clinical symptoms, the sensitivity and specificity of miRNAs as diagnostic indicators can be evaluated. Lai et al. showed that the highly expressed miRNAs miR-21, miR-146a, miR-155, miR-222, and miR-383 in mastitic milk showed 80% sensitivity and specificity, enabling their measurement to distinguish mastitic milk from normal milk [116]. A study by T Tzelos et al. revealed that the expression levels of bta-miR-223 and bta-miR-142-5p in milk led to their superior diagnostic performance (100% sensitivity, >81% specificity) in early inflammation assessments [117]. Although the samples analyzed in the two aforementioned studies comprised milk and not milk exosomes, the studies clearly demonstrated the value and potential of using miRNAs in milk for the early diagnosis of inflammation. In Table 2, the potential biomarkers in mammary epithelial cells, milk, blood, urine, and mammary tissue reported in the study are summarized, and the main types of markers were miRNAs.

## 5. Application Prospects of Exosomes as Drug Delivery Vehicles in Mastitis Treatment

Milk-derived exosomes have shown great promise in drug delivery and targeted therapy, and their value has been demonstrated in several studies in recent years. For example, studies have confirmed that milk-derived exosomes can be absorbed by a variety of cells [89,119] and can be used as delivery vehicles for short interfering RNA (siRNA), miRNA, and drugs [120,121]. The antiviral activity [122], anti-inflammatory activity [123,124,125], and the anticancer activity [126,127] of milk-derived exosomes have also been characterized through experiments. Several groups have compared the functional activity of bovine, buffalo, goat, and yak milk-derived exosomes [128,129,130,131]. As drug delivery vehicles, milk-derived exosomes present many advantages: safety as determined by failure to induce obvious immunogenicity or cytotoxicity [132,133,134]; ability to pass through barrier systems, such as the blood-brain barrier [135]; effective targeting [136], enhancement of the oral availability of drugs [137]; high stability against extreme stimuli [138,139]; standardization of cost and preparation processes [120,140,141], etc.

The biological activity of milk-derived exosomes has been demonstrated in a variety of diseases, but relatively little research has been conducted on their treatment in mastitis. Studies have shown that allogeneic umbilical cord blood mesenchymal stem cell-derived exosomes alleviated subclinical mastitis in dairy cows [142]. Anti-inflammatory activity and protection of milk fat barrier integrity has been exhibited by small-molecule compounds in mastitis in recent years [143,144,145]. The determination of whether the use of bovine milk exosomes as the delivery vehicle of these small-molecule compounds for the treatment of mastitis can increase the efficacy of treatments and whether it presents a reasonable solution to problems of drug resistance and antibiotic residues in clinical practice remain. These possibilities are worth considering and studying.

## 6. Exosome-Mediated Intercellular Communication Is Involved in the Occurrence and Development of Mastitis

**Figure 3:**
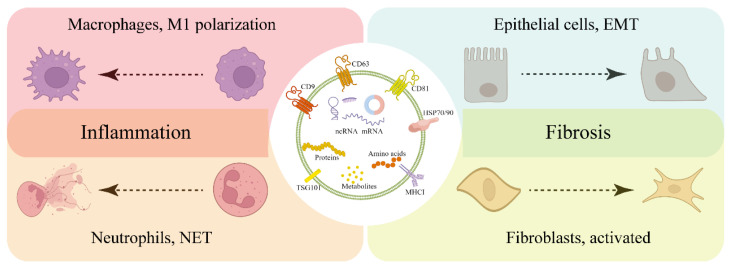
Mastitis-associated exosome-mediated intercellular communication. Mastitis-associated exosomes may mediate intercellular communication in various models of cell interactions, such as exosomes ingested by macrophages, and affect their M1-type polarization: ingested by neutrophils regulates its NET formation, ingested by epithelial cells promotes its EMT, and ingested by fibroblasts affects its activation.

Only by systematically understanding the pathogenesis of mastitis can the occurrence and development of dairy cow mastitis be effectively controlled. Exosomes mediate intercellular communication by transmitting signaling molecules taken up directly by recipient cells or by binding to ligands on the cell membrane. Single-cell sequencing of lactating mouse mammary gland cells revealed that ductal macrophages dominate the immune landscape of the mammary gland [146]. Macrophage function is to monitor mammary epithelial cells and promote tissue remodeling [147]. The results of a study by Cai et al. showed that exosomes generated after LTA stimulation of mammary epithelial cells (MAC-T and HC11 cells) promoted M1-type polarization of macrophages [98,99]. In addition, another study reported that TGF-β1-treated MAC-T cell-derived exosomes inhibited the proliferation of bovine macrophages [148]. These results suggest that exosome-mediated intercellular communication between mammary epithelial cells and macrophages may play an important role in mastitis and show the potential to be therapeutic targets. If macrophages are the “sentinels” of the immune system, then neutrophils are the “infantry” of the immune system. Neutrophils exert potent killing effects through degranulation, respiratory burst, and the formation of neutrophil extracellular traps [149]. In sepsis and acute lung injury, exosome-induced neutrophil activation plays an important role in tissue damage [150]. Evidence has suggested that neutrophils are abundant in mastitis tissue [151,152]. Therefore, the role played by mammary epithelial cell-derived exosome-mediated neutrophil activation in clinical mastitis deserves attention and research. An important feature of subclinical mastitis is mammary fibrosis. Fibroblasts are the main stromal cells in the mammary gland, and whether activation of fibroblasts or the EMT of epithelial cells is a key factor in mediating mammary fibrosis remains unknown. The results of a study by He et al. showed that SDF-1 secreted by primary mammary fibroblasts stimulated by LTA may promote the occurrence of fibrosis by inducing the EMT if mammary epithelial cells [153]. While mammary epithelial cells regulate the function of surrounding cells by exerting paracrine effects, a bystander effect may be induced by bacterial infection. That is, exosomes secreted by mammary epithelial cells stimulated by bacterial virulence factors may be absorbed by other mammary epithelial cells in the mammary gland, thereby spreading infection and activating an inflammatory response (Figure 3). In fact, little is known about the contribution of exosome-mediated intercellular communication in mastitis, and further exploration and research are still needed.

## 7. Challenges of Using Exosomes in the Diagnosis and Treatment of Mastitis

Recent studies have demonstrated the potential of milk-derived exosome use in diagnostics, therapy, and drug delivery. However, many problems remain to be solved before exosomes can be used to treat mastitis in the clinic. First, pretreatment and storage of milk present challenges; that is, improper handling affects the quantity, purity, and biological activity of exosomes, and residual harmful substances can compromise the safety of subsequent applications. Second, the method used to isolate milk exosomes needs be selected comprehensively considering the amount of exosomes required, technical difficulty of the process, hardware requirements, cost, and level of purity for subsequent applications. One study compared unpasteurized human and bovine milk pretreatment, exosome isolation, and RNA extraction methods [154], and suggested that the combined application of different principles and methods may be the most applicable strategy for obtaining exosomes. Establishing a standardized processing method based on the two aforementioned challenges will greatly promote the industrialization and commercial application of milk-derived exosomes. Third, the drug-loading rate and targeting efficacy of milk-derived exosomes have limited their therapeutic applications. Engineering exosomes or combining exosomes with other biological organic materials have been the recent research direction [155]. Fourth, recent research on exosomes related to mastitis has mainly focused on miRNAs. The possible reasons for this focus related to miRNAs being highly conserved among species is their abundance in exosomes and the predictability of downstream effects. However, based on omics technologies, such as metabolomics and proteomics, lncRNAs, proteins, and metabolites in exosomes have great value and should be mined. Fifth, the development and application of clinical and subclinical mastitic predictive and diagnostic biomarkers are needed. Some of the biomarkers identified in recent research show problems, such as fragmentation, lack of integration, unsuitable for clinical practice, and lack of sensitivity or specificity in large groups. These factors place the potential biomarkers identified by numerous studies in the theoretical stage, and their value in clinical diagnostics remains unclear. Sixth, identifying the sources of exosomes in milk is a problem limiting the functional exploration and application of milk-derived exosomes. Single-cell sequencing technology is a powerful tool for solving this problem. Seventh, the sorting mechanism of mastitis-related exosome cargo and the molecular mechanism mediating intercellular communication remain unclear; these areas of inquiry in mastitis research are still in their infancy. Three atlas-based articles describe the m6A modification of mRNA and circular RNA (circRNA) in MAC-T cells after *E. coli* or *S. aureus* infection [156,157,158]. Considering the function of m6A modification in promoting miRNA maturation [159], study into the role played by m6A in mastitis exosomal cargo sorting is warranted. In addition, RNA-binding proteins mediate miRNA loading by recognizing specific motifs and also deserve attention.

In conclusion, exosomes exhibit important application potential for the prediction, diagnosis, and treatment of mastitis. Further experimental studies on exosomes will deepen our understanding of the pathogenesis of mastitis, and carrying out prospective clinical trials will promote the value of exosomes in the clinical application to mastitis.

## Figures and Tables

**Table 1:** Research Status of Exosomes in Mastitis.

Source of Exosomes	Isolation Method	Identification Method	Key Results	Ref
BMEC	Ultracentrifugation	TEM + WB	Identified protein species in normal exosomes	[90]
MAC-T	Ultracentrifugation	TEM + NTA + FCM	Constructed mRNA and LncRNA expression profiles of normal and *S. aureus*-infected MAC-T cell-derived exosomes	[91]
MAC-T	Ultracentrifugation	TEM + NTA	Constructed mRNA expression profiles of selenium-pretreated normal and *S. aureus*-infected MAC-T cell-derived exosomes	[97]
MAC-T and milk	Ultracentrifugation	TEM + NTA + WB	MAC-T cell-derived and milk-derived exosomes showed similar miRNA and protein expression profiles	[89]
HC11	Ultracentrifugation	TEM + NTA + WB	HC11 cell-derived exosome miR-211 induces macrophages M1-type polarization by targeting SOCS1	[98]
MAC-T	Ultracentrifugation	TEM + NTA + WB	MAC-T cell-derived exosomes induce macrophages M1-type polarization	[99]
MAC-T	Ultracentrifugation	TEM + NTA + FCM	MAC-T cell-derived exosomal lnc-AFTR plays a regulatory role in apoptosis and inflammation induced by *S. aureus* by mediating FAS degradation	[100]
MAC-T	Ultracentrifugation	TEM + WB	MAC-T cell-derived exosome miR-193b-5p promotes inflammatory response	[101]
Milk	Sucrose Gradient Centrifugation + Filtration Purification	-	Constructed miRNA expression profiles of milk exosomes after *Staphylococcus* spp. infection, and bta-miR-142-5p and bta-miR-223 were initially identified as potential early detection markers	[75]
Milk	Ultracentrifugation	TEM + NTA + WB	The results showed that 18 miRNAs were differentially expressed between the control and *S. aureus*-infected groups and revealed that bta-miR-142-5p and bta-miR-223 may play a role in mastitis	[76]
Milk	Ultracentrifugation	TEM + NTA + WB	Analysis of the results of 3 consecutive days of sampling revealed the stability of miRNAs within the exosomes, and bta-miR-223-3p was found to be significantly upregulated in subclinical mastitis	[78]
Milk	Ultracentrifugation	TEM + NTA + FCM	Constructed exosome miRNA expression profiles for normal and *S. aureus*-infected groups	[77]

-: Indicates that this part of the information is missing from the paper.

**Table 2:** Potential Biomarkers in Mastitis.

Sample	Types of Mastitis	Key Molecule	Expression	Ref
Peripheral Blood	Subclinical mastitis	bta-miR-21/bta-miR-146a/bta-miR-155/bta-miR-222/bta- miR-383	UP	[116]
Peripheral Blood	Subclinical mastitis	bta-miR-223/bta-miR-142-5p	UP	[117]
Peripheral Blood and Milk	Subclinical mastitis (*S. aureus*)	PON1	DOWN	[115]
Peripheral Blood	Subclinical mastitis (*S. aureus*)	bta-miR-21	UP	[103]
Peripheral Blood	Mastitis	bta-miR-1301	UP	[104]
Peripheral Blood	Subclinical mastitis (*S. aureus*)	bta-miR-2284r	DOWN	[104]
Peripheral Blood	Subclinical mastitis	Valine (Val)/serine (Ser)/tyrosine (Tyr)/phenylalanine (Phe)/isoleucine (Ile)	-	[106]
Peripheral Blood	Mastitis	Nonesterified fatty acid/aspartate aminotransferase	UP	[118]
Peripheral Blood	Subclinical mastitis	Lysine/leucine/isoleucine/kynurenine/sphingomyelin (SM) C26:0/Ornithine/lysoPC A C17:0/SM C26:1	-	[107]
Urine	Subclinical mastitis	Acylcarnitines (ACs)/phosphatidylcholines (PCs)/amino acids (AAs)/biogenic amines (BAs)	-	[108]
Milk	Subclinical mastitis (*S. aureus*)	bta-miR-142-5p/bta-miR-223	UP	[75,76]
Milk	Subclinical mastitis	bta-miR-223-3p	UP	[78]
Milk	Subclinical mastitis *(S. aureus*)	bta-miR-378/bta-miR-185	UP	[77]
Bovine Mammary Gland Tissue	Clinical mastitis (*E. coli*)	bta-miR-202/bta-miR-2357	UP	[111]
Bovine Mammary Gland Tissue	Subclinical mastitis (*S. aureus*)	bta-miR-7863	UP	[111]
Bovine Mammary Gland Tissue	Subclinical mastitis (*S. agalactiae*)	bta-miR-223	UP	[112]
Bovine Mammary Gland Tissue	Subclinical mastitis (*S. agalactiae*)	bta-miR-26a	DOWN	[112]
Bovine Mammary Gland Tissue	Subclinical mastitis (*S. aureus*)	bta-miR-223/bta-miR-132/bta-miR-1246	UP	[109]
HC11 cells	LTA	mmu-miR-211	UP	[98]
MAC-T cells	*S. aureus*	Lnc-AFTR	DOWN	[100]
MAC-T cells	LPS	bta-miR-193b-5p	UP	[101]

## Abbreviations

**Abbreviation**	** Full name**
AD	Alzheimer’s disease
AKI	Acute kidney injury
ALIX	Apoptosis-linked gene 2-interacting protein X
BMECs	Bovine mammary epithelial cells
circRNA	Circular RNA
E. coli	Escherichia coli
EMT	Epithelial-mesenchymal transition
ESCRT	Endosomal sorting complexes required for transport
FCM	Flow Cytometry
GC-MS	Gas Chromatography-Mass Spectrometer
HC11	Mouse Mammary Epithelial Cell Lines-HC11
HRS	Hepatocyte growth factor-regulated tyrosine kinase substrate
IBD	Inflammatory bowel disease
ISEV	International Society for Extracellular Vesicles
IVLs	Internal intraluminal vesicles
LC-MS	Liquid Chromatography-Mass Spectrometry
LncRNAs	Long noncoding RNAs
LPS	Lipopolysaccharide
LTA	Lipoteichoic acid
M1	Macrophages type I
M2	Macrophages type II
m6A	N6-Methyladenosine
MAC-T	Bovine Mammary Epithelial Cell Lines-MAC-T
MAPK	Mitogen-activated protein kinase
MVBs	Multivesicular bodies
NET	Neutrophil extracellular traps
NF-κB	Nuclear factor kappa B
nSMase2	Neutral sphingomyelinase2
NTA	Nanoparticle Tracking Analyzer
PD	Parkinson’s disease
PEG	Polymer polyethylene glycol
PON1	Paraoxonase 1
S. agalactiae	Streptococcus agalactiae
S. Aureus	Staphylococcus aureus
SCC	Somatic cell count
siRNA	Short interfering RNA
SNARE	Soluble N-ethylmaleimide-sensitive factor (NSF) attachment protein receptor
STAM1	Signal transducing adapter molecule 1
TEM	Transmission Electron Microscopy
TSG101	Tumor susceptibility gene 101
WB	Western blot
